# Insight into the Strong Antioxidant Activity of Deinoxanthin, a Unique Carotenoid in *Deinococcus Radiodurans*

**DOI:** 10.3390/ijms11114506

**Published:** 2010-11-10

**Authors:** Hong-Fang Ji

**Affiliations:** Shandong Provincial Research Center for Bioinformatic Engineering and Technique, Shandong University of Technology, Zibo 255049, China; E-Mail: jhf@sdut.edu.cn; Tel.:+86-533-278-0271; Fax: +86-533-278-0271

**Keywords:** deinoxanthin, lowest triplet excitation energy, bond dissociation enthalpy, density functional theory

## Abstract

Deinoxanthin (DX) is a unique carotenoid synthesized by *Deinococcus radiodurans*, one of the most radioresistant organisms known. In comparison with other carotenoids, DX was proven to exhibit significantly stronger reactive oxygen species (ROS)-scavenging activity, which plays an important role in the radioresistance of *D. radiodurans*. In this work, to gain deeper insights into the strong antioxidant activity of DX, the parameters characterizing ROS-scavenging potential were calculated by means of quantum chemical calculations. It was found that DX possesses lower lowest triplet excitation energy for its unique structure than other carotenoids, such as β-carotene and zeaxanthin, which endows DX strong potential in the energy transfer-based ROS-scavenging process. Moreover, the H-atom donating potential of DX is similar to zeaxanthin according to the theoretical homolytic O-H bond dissociation enthalpy. Thus, the large number of conjugated double bonds should be crucial for its strong antioxidant activity.

## 1. Introduction

**Figure 1.**
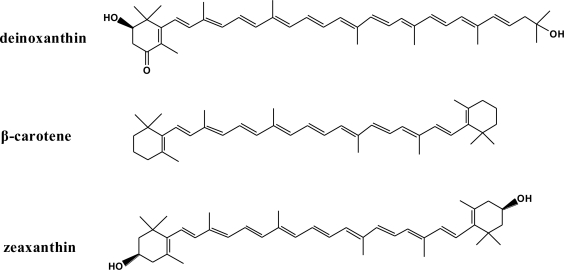
Chemical structures of deinoxanthin, β-carotene and zeaxanthin.

*Deinococcus radiodurans* is a red-pigmented, nonphotosynthetic bacterium well known for its resistance to ionizing radiation [1–3]. It has been demonstrated that cellular antioxidants make important contributions to the radioresistance of *D. radiodurans* besides the efficient and accurate DNA repair strategy [4,5]. Among nonenzymic antioxidants, carotenoids possess efficient reactive oxygen species (ROS) scavenging capacity [6]. It is interesting to note that *D. radiodurans* synthesizes a unique ketocarotenoid, deinoxanthin (DX, Figure 1), as its major carotenoid [7–9]. DX was proven to exhibit significantly stronger ROS scavenging ability than other known carotenoids, such as β-carotene (BC, Figure 1) and zeaxanthin (ZX, Figure 1) [9], and the strong antioxidant effect of DX plays an important role in the radioresistance of *D. radiodurans* [9]. Therefore, it is interesting to explore the mechanistic underpinnings underlying the higher antioxidant potential of DX relative to other carotenoids. In the present work, by means of quantum chemical calculations, the parameters to characterize the antioxidant potential of DX, including the lowest triplet excitation energy (E_T1_) and homolytic O-H bond dissociation enthalpy (BDE), were estimated. The theoretical results further our understanding of the higher ROS-scavenging activities of DX compared to BC and ZX.

## 2. Calculation Methods

The structures of DX, BC and ZX were fully optimized by hybrid density functional theory (DFT) [10,11] and B3LYP [12–14] functional with 6–31G(d) Gaussian basis set. The nature of the stationary point was ascertained by performing harmonic frequency calculations. The lowest triplet state energies (E_T1_s) of DX, BC and ZX were calculated by time-dependent DFT (TD-DFT) formalism [15–17] with the same basis set. To ensure the accuracy of the results, the O-H BDEs of DX and ZX were estimated using a combined method labeled as (RO)B3LYP/6-311+G(2d,2p)//AM1/AM1, which takes advantage of accuracy and economy [18–21]. As the hydroxyls of DX and ZX are not conjugated with the polyene chain, which should influence little on the O-H bond dissociation reactions, only one double bond in the polyene chain is reserved while the rest was replaced by a methyl when estimating the O-H BDEs of DX and ZX. The two hydroxyl groups of ZX are equivalent and only one is considered. The O-H BDE was estimated according to the following equation, O-H BDE = *H*_r_ + *H*_h_ – *H*_p_ [18–21], in which, *H*_r_ is the enthalpy of radical generated through H-abstraction reaction, *H*_h_ is the enthalpy of H-atom, −0.49765 hartree, and *H*_p_ is the enthalpy of parent molecule.

All calculations were performed using Gaussian 03 package of programs [22].

## 3. Results and Discussion

Carotenoids are efficient singlet oxygen (^1^O_2_) quenchers. Owing to the rather low E_T1_ of carotenoids, ^1^O_2_ can be quenched through energy transfer (Equation 1); generating triplet excited state carotenoids and ground state oxygen (^3^O_2_).

(1) $$\text{Car}({\text{S}}_{0})+{}^{1}{\text{O}}_{2}\to \text{Car}({\text{T}}_{1})+{}^{3}{\text{O}}_{2}$$

The ^1^O_2_ quenching capability is a good indicator of the E_T1_. Table 1 lists the TD-B3LYP/6-31G(d) estimated E_T1_ of DX, BC and ZX. The theoretically predicted E_T1_s of BC and ZX are close to the experimental value [23,24], which verifies the methodology.

The E_T1_s of the three carotenoids are lower than the deactivation energy of ^1^O_2_ (0.97 eV), which indicates that they are ^1^O_2_ quenchers. Moreover, the E_T1_ of DX is approximately 0.1 eV lower relative to those of BC and ZX. The lower E_T1_ of DX will make the energy transfer process more favorable energetically, relative to the other two carotenoids, which is consistent with the experimental finding that DX possesses stronger ^1^O_2_ quenching ability than BC and ZX [9]. Moreover, it was reported that the ^1^O_2_ quenching enhanced as the number of conjugated double bonds in the polyene chain of carotenoids increased, by examining various naturally occurring carotenoids [25]. Thus, it can be inferred that the higher ^1^O_2_ quenching ability of DX than that of other carotenoids should mainly arise from its extended conjugated double bonds system.

The direct H-atom transfer is one of the most important radical-scavenging processes [18–21]. Taking RO· as an example, the direct H-atom transfer process can be represented as follows.

(2) CarOH + RO⋅ → CarO⋅ + ROH

Among the three carotenoids, DX and ZX possess hydroxyls as potential H-atom donors in their structures. O-H BDE acts as an appropriate parameter to characterize the H-atom donating ability [18–21]. The O-H BDE of the hydroxyl in the six-membered ring of DX is calculated to be about 101.92 kcal/mol, and that of the butyl hydroxyl at the end of the polyene chain is about 104.71 kcal/mol. This indicates that on thermodynamic grounds the hydroxyl in the six-membered ring should play a predominant role in the H-atom transfer-based ROS scavenging processes of DX. Moreover, the theoretical O-H BDE of ZX is about 101.74 kcal/mol. The close O-H BDEs between DX and ZX imply that they possess similar H-atom donating potential thermodynamically.

Collectively, the extended conjugated double bonds system of DX seems crucial for its strong ROS-scavenging activity. The larger number of conjugated double bonds means DX possesses lower E_T1_, and thus higher ^1^O_2_ quenching potential through energy transfer, in comparison with BC and ZX. The strong ROS scavenging ability of DX renders this unique carotenoid great potential in antioxidant therapy application.

## Figures and Tables

**Table 1.** Theoretically estimated lowest triplet excitation energies (E_T1_) of deinoxanthin (DX), β-carotene (BC) and zeaxanthin (ZX) (in eV).

	**DX**	**BC**	**ZX**
Theoretical data	0.69	0.80	0.80
Experimental data	-	0.88 [23]	0.87 [24]

## References

[1] Daly MJ, Minton KW (1995). Interchromosomal recombination in the extremely radioresistant bacterium *Deinococcus radiodurans*. J. Bacteriol.

[2] Cox MM, Battista JR (2005). *Deinococcus radiodurans*—the consummate survivor. Nat. Rev. Microbiol.

[3] Slade D, Lindner AB, Paul G, Radman M (2009). Recombination and replication in DNA repair of heavily irradiated *Deinococcus radiodurans.*. Cell.

[4] Ghosal D, Omelchenko MV, Gaidamakova EK, Matrosova VY, Vasilenko A, Venkateswaran A, Zhai M, Kostandarithes HM, Brim H, Makarova KS, Wackett LP, Fredrickson JK, Daly MJ (2005). How radiation kills cells: survival of *Deinococcus radiodurans* and *Shewanella oneidensis* under oxidative stress. FEMS Microbiol. Rev.

[5] Markillie LM, Varnum SM, Hradecky P, Wong K (1999). Targeted mutagenesis by duplication insertion in the radioresistant bacterium *Deinococcus radiodurans*: radiation sensitivities of catalase (*kat*A) and superoxide dismutase (*sod*A) mutants. J. Bacteriol.

[6] Paiva SA, Russell RM (1999). Beta-carotene and other carotenoids as antioxidants. J. Am. Coll. Nutr.

[7] Lemee L, Peuchant E, Clerc M, Brunner M, Pfander H (1997). Deinoxanthin: A new carotenoid isolated from *Deinococcus radiodurans.*. Tetrahedron.

[8] Saito T, Ohyama Y, Ide H, Ohta S, Yamamoto O (1998). A carotenoid pigment of the radioresistant bacterium *Deinococcus radiodurans.*. Microbios.

[9] Tian B, Xu Z, Sun Z, Lin J, Hua Y (2007). Evaluation of the antioxidant effects of carotenoids from *Deinococcus radiodurans* through targeted mutagenesis, chemiluminescence, and DNA damage analyses. Biochim. Biophys. Acta.

[10] Hohenberg P, Kohn W (1964). Inhomogeneous electron gas. Phys. Rev.

[11] Kohn W, Sham LJ (1965). Self-consistent equations including exchange and correlation effects. Phys. Rev.

[12] Lee C, Yang W, Parr RG (1988). Development of the Colle-Salvetti correlation energy formula into a functional of the electron density. Phys. Rev. B.

[13] Becke AD (1993). A new mixing of Hartree-Fock and local density-functional theories. J. Chem. Phys.

[14] Stephens PJ, Devlin FJ, Chabalowski CF, Frisch MJ (1994). *Ab Initio* calculation of vibrational absorption and circular dichroism spectra using density functional force fields. J. Phys. Chem.

[15] Stratmann RE, Scuseria GE, Frisch MJ (1998). An efficient implementation of time-dependent density-functional theory for the calculation of excitation energies of large molecules. J. Chem. Phys.

[16] Bauernschmitt R, Ahlrichs R (1996). Treatment of electronic excitations within the adiabatic approximation of time dependent density functional theory. Chem. Phys. Lett.

[17] Casida ME, Jamorski C, Casida KC, Salahub DR (1998). Molecular excitation energies to high-lying bound states from time-dependent density-functional response theory: characterization and correction of the time-dependent local density approximation ionization threshold. J. Chem. Phys.

[18] Zhang HY (2005). Structure-activity relationships and rational design strategies for radical-scavenging antioxidants. Curr. Comput. Aided Drug Des.

[19] Wright JS, Johnson ER, DiLabio GA (2001). Predicting the activity of phenolic antioxidants: theoretical methods, analysis of substituent effects, and application to major families of antioxidants. J. Am. Chem. Soc.

[20] Ji HF, Zhang HY, Shen L (2006). A theoretical elucidation of radical-scavenging power of cyanindin. Nat. Prod. Commun.

[21] Ji HF, Zhang HY, Shen L (2006). Proton dissociation is important to understanding structure-activity relationships of gallic acid antioxidants. Bioorg. Med. Chem. Lett.

[22] Frisch MJ, Trucks GW, Schlegel HB, Scuseria GE, Robb MA, Cheeseman JR, Montgomery JA, Vreven T, Kudin KN, Burant JC, Millam JM, Iyengar SS, Tomasi J, Barone V, Mennucci B, Cossi M, Scalmani G, Rega N, Petersson GA, Nakatsuji H, Hada M, Ehara M, Toyota K, Fukuda R, Hasegawa J, Ishida M, Nakajima T, Honda Y, Kitao O, Nakai H, Klene M, Li X, Knox JE, Hratchian HP, Cross JB, Adamo C, Jaramillo J, Gomperts R, Stratmann RE, Yazyev O, Austin AJ, Cammi R, Pomelli C, Ochterski JW, Ayala PY, Morokuma K, Voth GA, Salvador P, Dannenberg JJ, Zakrzewski VG, Dapprich S, Daniels AD, Strain MC, Farkas O, Malick DK, Rabuck AD, Raghavachari K, Foresman JB, Ortiz JV, Cui Q, Baboul AG, Clifford S, Cioslowski J, Stefanov BB, Liu G, Liashenko A, Piskorz P, Komaromi I, Martin RL, Fox DJ, Keith T, Al-Laham MA, Peng CY, Nanayakkara A, Challacombe M, Gill PMW, Johnson B, Chen W, Wong MW, Gonzalez C, Pople JA (2003). Gaussian 03.

[23] Haley JL, Fitch AN, Goyal R, Lambert C, Truscott TG, Chacon JN, Stirling D, Schalch W (1992). The S_1_ and T_1_ energy levels of all-trans-.-carotene. J Chem Soc Chem Commun.

[24] Wang C, Tauber MJ (2010). High-yield singlet fission in a zeaxanthin aggregate observed by picosecond resonance Raman spectroscopy. J. Am. Chem. Soc.

[25] Hirayama O, Nakamura K, Hamada S, Kobayasi Y (1994). Singlet oxygen quenching ability of naturally occurring carotenoids. Lipids.

